# Salidroside May Attenuate Palmitic Acid-Induced Bovine Oocyte Impairment Associated with Mitochondrial and Antioxidant Restoration

**DOI:** 10.3390/antiox15101254

**Published:** 2026-10-01

**Authors:** Shu-Xian Guo, Zhi-Qiang Feng, Run-Bo Li, Fan Zhao, Yan-Hua Li, Wang Han, Jian-Yong Han, Zhi-Shen Mu, Su-Ying Cao

**Affiliations:** 1 Animal Science and Technology College, Beijing University of Agriculture, Beijing 102206, China; 202530321127@bua.edu.cn (S.-X.G.);; 2 State Key Laboratory of Animal Biotech Breeding, Frontiers Science Center for Molecular Design Breeding (MOE), College of Biological Sciences, China Agricultural University, Beijing 100193, China; b20233020145@cau.edu.cn (Z.-Q.F.); hanjy@cau.edu.cn (J.-Y.H.); 3 Beijing Dairy Cattle Center, Beijing 100192, China; 4 Global Research and Innovation Center, Inner Mongolia Mengniu Dairy (Group) Co., Ltd., Inner Mongolia Key Laboratory of Dairy Nutrition, Health and Safety, Hohhot 011517, China

**Keywords:** palmitic acid, Salidroside, cows, oocyte, mitochondrial, stress

## Abstract

Negative energy balance (NEB) in postpartum dairy cows elevates systemic non-esterified fatty acid (NEFA) levels, including palmitic acid (PA), which impair oocyte quality and contribute to reduced reproductive performance. Salidroside (SAL) has attracted attention for its prominent role in maintaining mitochondrial homeostasis. However, whether SAL can alleviate PA-induced oocyte damage remains unclear. Therefore, the present study was designed to investigate the protective effects of SAL against PA-induced oocyte damage and to explore the underlying mechanisms. We found that 0.2 mM PA triggered oxidative stress in oocytes, as evidenced by fluorescent staining results showing elevated ROS levels, reduced GSH content, and single-cell transcriptomic data revealing transcriptional suppression of *HSPE1*, *ROMO1*, and *GSTM3*. Meanwhile, PA treatment impaired mitochondrial function, reflected by decreased mitochondrial membrane potential and ATP levels, along with significant downregulation of mitochondrial function related genes, including *NDUFA4*, *NDUFA6*, *ATP5PO*, *ATP5ME*, *COX5A*, and *UQCRH*. These changes help explain why PA exposure reduces oocyte maturation rate and embryonic developmental potential. Notably, supplementation with 5 μM SAL effectively reversed the PA induced damage and restored oocyte developmental competence to control level to levels comparable to untreated controls. These findings suggest that SAL can restore oocyte developmental potential by mitigating the lipotoxic microenvironment induced by PA administration.

## 1. Introduction

In vitro embryo production (IVP) can accelerate the multiplication of genomically selected elite donors, rapidly translating high genetic merit into offspring, but its current efficiency in dairy cattle remains relatively low, which is largely attributable to impaired oocyte quality [1,2,3,4]. Most high-producing dairy cows experience a negative energy balance (NEB) after calving to adapt to milk production. To meet energy demands, dairy cows mobilize substantial amounts of non-esterified fatty acids (NEFA) to the liver for energy production [5]. NEFA concentration in blood rapidly equilibrates with follicular fluid, exposing cumulus–oocyte complexes to a lipotoxic microenvironment that adversely affects oocyte quality [6,7,8]. Palmitic acid (PA) is the predominant saturated NEFA in both circulation and follicular fluid during negative energy balance, serving as a primary substrate for mitochondrial β-oxidation [6,9,10]. However, excessive PA triggers oxidative stress in oocytes, leading to a decline in mitochondrial membrane potential and impairment of respiratory capacity, which in turn compromises oocyte developmental potential and ultimately impairs embryonic production efficiency [7,11,12]. Therefore, identifying intervention strategies capable of effectively alleviating PA-induced oocyte damage has become a critical step for improving IVP efficiency based on oocytes collected from dairy cows.

Salidroside (SAL) is a phenolic compound isolated from Rhodiola plants. It exhibits a wide range of pharmacological activities, including anti-inflammatory, antioxidant, antitumor, anti-aging, anti-hypoxic, and mitochondria-protective effects [13,14,15]. In recent years, its potential value in improving oocyte quality has attracted growing attention. Liu reported that SAL supplementation significantly increased oocyte maturation and blastocyst rates, while reducing ROS levels and increasing GSH content, indicating that SAL effectively alleviates oxidative stress-induced damage to oocytes [16]. In mouse models, SAL also demonstrated notable mitochondrial protective effects. It not only improved oocyte quality in reproductively aged mice by enhancing mitophagy, but also delayed post-ovulatory oocyte aging and restored mitochondrial distribution, membrane potential, and ATP content [17]. Consistent with these findings, Gu showed that intraperitoneal injection of SAL significantly improved reproductive function in aged mice, further confirming its capacity to enhance mitochondrial function [18]. Although these studies have revealed the potential of SAL in protecting oocyte quality, whether SAL can alleviate PA-induced oxidative stress to bovine oocytes and the underlying molecular mechanisms remain largely unknown.

Here, we established a bovine oocyte model of PA-induced lipotoxicity to test the hypothesis that SAL protects against PA-triggered oocyte damage by enhancing mitochondrial function and alleviating oxidative stress, and to elucidate the underlying mechanisms. By integrating analyses of oocyte maturation quality, embryonic developmental competence, single-cell transcriptomic profiles, and mitochondrial function alongside oxidative stress responses, we sought to determine whether and how SAL reverses PA-induced oocyte injury. These efforts are expected to provide a theoretical foundation for the use of SAL in safeguarding oocyte quality under lipotoxic conditions.

## 2. Materials and Methods

Unless otherwise indicated, all reagents employed in the present study were purchased from Sigma-Aldrich (St. Louis, MO, USA). Culture medium (TCM199) and fetal bovine serum (FBS) were acquired from Gibco (Life Technologies Inc., Grand Island, NY, USA). All experimental procedures in this study strictly complied with the guidelines for animal welfare and ethics, and were formally approved by the Animal Ethics Committee.

### 2.1. In Vitro Maturation (IVM)

Ovaries were collected from a local slaughterhouse and transported to the laboratory in pre-warmed (30 °C) physiological saline within 2 h. Cumulus–oocyte complexes (COCs) were aspirated from follicles of 2–8 mm diameter. Under a stereomicroscope, COCs with 2–3 layers of compact cumulus cells were selected and randomly allocated to the following experimental groups: (1) control (basal maturation medium only); (2) PA treatment groups: 0.2 mM or 0.4 mM palmitic acid (KC002, Kunchuang Biotechnology, Xi’an, China); (3) SAL treatment groups: 1, 5, 10, 20, or 40 μM salidroside (HY-N0109, MedChemExpress, Monmouth Junction, NJ, USA); and (4) combination group: 0.2 mM palmitic acid plus 5 μM salidroside. The COCs were incubated at 38.5 °C under 5% CO_2_ and saturated humidity for 22–24 h. The basal in vitro maturation (IVM) medium consisted of TCM-199 (Gibco, 11150-059) supplemented with 0.01 IU/mL FSH, 0.01 IU/mL LH, 1 μg/mL estradiol, 10 μg/mL heparin, 10% (*v*/*v*) fetal bovine serum (FBS), and 1% antibiotic solution (100 U/mL penicillin and 100 μg/mL streptomycin).

### 2.2. In Vitro Fertilization (IVF)

COCs (15–20 per group) were transferred into 50 μL droplets of Brackett and Oliphant (BO) fertilization medium [19] supplemented with 10 μg/mL heparin and 4 mg/mL fatty-acid-free BSA. Frozen semen was thawed at 38.5 °C for 30 s, and then washed twice in BO medium containing 10 mM caffeine and 4 mg/mL fatty-acid-free BSA by centrifugation at 623 *g* for 5 min. The resulting sperm pellet was resuspended to a final concentration of 1 × 10^6^ cells/mL. A 50 μL aliquot of the sperm suspension was added to each fertilization droplet, and the gametes were co-incubated at 38.5 °C in a 5% CO_2_ atmosphere with saturated humidity for 18 h.

### 2.3. In Vitro Embryo Production (IVP)

At 18 h post-fertilization, residual cumulus cells and spermatozoa were removed from the oocytes by gentle pipetting. The presumptive zygotes were then cultured in vitro culture (IVC) medium (Bioscience, BOIVC2501) at a density of 20–25 zygotes per group. On day 2 post-fertilization, cleaved embryos were selected and transferred to fresh IVC medium for further 5 days of culture. Cleavage and blastocyst rates were recorded on days 2 and 7 post-fertilization, respectively.

### 2.4. Evaluation of Nuclear and Cytoplasmic Maturation in Oocytes

To evaluate the nuclear maturation status of oocytes, cumulus cells were removed using 0.1% hyaluronidase. Oocytes that had extruded the first polar body were selected under a stereomicroscope, and the first polar body extrusion rate was calculated accordingly.

Cytoplasmic maturation was further evaluated by assessing cortical granule distribution and F-actin polymerization. After removal of cumulus cells, oocytes were treated with 0.5% pronase at 37 °C for 5 min to remove the zona pellucida, fixed in 4% paraformaldehyde for 30 min, and permeabilized with 0.5% Triton X-100 for 15 min.

Cortical granule staining: The fixed oocytes were incubated in DPBS containing 3 mg/mL BSA and 7.5 mg/mL glycine for 15 min to block non-specific binding, and then stained with 20 µg/mL FITC-conjugated peanut agglutinin (FITC-PNA) in the dark at room temperature for 30 min. After staining, images were captured using a confocal laser scanning microscope (TCS SP8, Leica, Wetzlar, Germany) equipped with a plan apochromat 20×/0.75 NA objective lens (HC PL APO 20×/0.75 CS2), with identical settings across all experimental groups (same below).

F-actin staining: The fixed oocytes were blocked with DPBS containing 1% BSA for 30 min, and then stained with Alexa Fluor Plus 555-conjugated phalloidin (Invitrogen, CA, USA) in the dark at room temperature for 60 min. After staining, images were captured using the same confocal laser scanning microscope with identical acquisition parameters across all experimental groups.

Quantification was performed using ImageJ (version 1.54f) software (NIH, Bethesda, MD, USA). For cortical granule fluorescence intensity quantification, each oocyte was outlined as circle A, and a concentric circle B was drawn in the region of lower fluorescence intensity inside the oocyte. The average fluorescence intensity of the outer cortical region was calculated using the following formula: Average fluorescence intensity of the outer region = (Total fluorescence intensity of A − Total fluorescence intensity of B)/(Area of A − Area of B). For F-actin fluorescence intensity analysis, the same concentric circle method was applied. The average values from multiple oocytes were used to represent the final fluorescence intensity for each treatment group.

### 2.5. Immunofluorescence Staining

Embryos were fixed with 4% paraformaldehyde at room temperature for 30 min, then transferred to washing solution (0.1% PVA/DPBS,) and washed three times (5 min each) on an orbital shaker at 120 rpm. Subsequently, embryos were permeabilized with 0.5% Triton X-100 at room temperature for 15 min, followed by three additional washes (5 min each) in washing solution under the same conditions. Blocking was performed with washing solution containing 3% BSA on an orbital shaker at 80 rpm for 1 h. Then, embryos were incubated with primary antibodies diluted in blocking solution according to the manufacturer’s recommendations at 4 °C overnight with gentle agitation. The next day, embryos were washed three times (5 min each) in washing solution on an orbital shaker at 100 rpm. Afterward, embryos were incubated with species-specific secondary antibodies diluted in washing solution for 1 h at room temperature in the dark with shaking at 50 rpm, followed by three washes (5 min each) in washing solution under dark conditions on an orbital shaker at 120 rpm. Finally, nuclei were stained with DAPI (1:10,000 dilution) for 3 min at room temperature in the dark with gentle shaking. After staining, embryos were ready for fluorescence observation. SOX2 and CDX2 are well-established lineage markers for the inner cell mass (ICM) and trophectoderm (TE), respectively, and are widely used for blastocyst quality assessment in mammalian species. The distribution of ICM and TE cells in blastocysts was assessed based on the immunofluorescence intensities of SOX2 (Santa Cruz Biotechnology, Santa Cruz, CA, USA, sc-365823) and CDX2 (PeproTech, Cranbury, NJ, USA, 82659-1-RR), respectively.

### 2.6. Measurement Reactive Oxygen Species (ROS) and Glutathione (GSH) Levels in Oocytes

Oocytes after 22 h of IVM were treated with 0.1% hyaluronidase and gently pipetted to remove cumulus cells. After denudation, oocytes were washed three times with 0.1% PVA/DPBS and then subjected to ROS and GSH fluorescence staining, respectively.

Intracellular ROS levels were measured using the DCHFDA fluorescent probe. Cumulus-denuded oocytes (10–15 per group) were incubated with 100 μM DCHFDA at 37 °C in the dark for 30 min. After three washes with 0.1% PVA/DPBS, fluorescence signals were captured under a fluorescence microscope and quantified with ImageJ software. For each oocyte, a circle was drawn to outline the entire fluorescent region of the oocyte, and the mean fluorescence intensity per unit area was calculated. The average values from multiple oocytes were used to represent the final fluorescence intensity for each treatment group.

Intracellular GSH levels were measured using the ThiolTracker™ Violet fluorescent probe. Cumulus-denuded oocytes (10–15 per group) were incubated with 20 μM ThiolTracker™ Violet (Invitrogen/Molecular Probes, Carlsbad, CA, USA) at 37 °C in the dark for 30 min. After three washes with 0.1% PVA/DPBS, fluorescence signals were captured under a fluorescence microscope and quantified with ImageJ software. For each oocyte, a circle was drawn to outline the entire fluorescent region of the oocyte, and the mean fluorescence intensity per unit area was calculated. The average values from multiple oocytes were used to represent the final fluorescence intensity for each treatment group.

### 2.7. Measurement of Mitochondrial Membrane Potential (MMP)

Mitochondrial membrane potential (MMP) was evaluated using a JC-1 assay kit (Solarbio, Beijing, China) according to the manufacturer’s recommended protocol. Briefly, cumulus-denuded oocytes (10–15 per group) were incubated with 4 μM JC-1 working solution at 38.5 °C in the dark for 30 min. After incubation, oocytes were washed three times with 0.1% PVA/DPBS and immediately observed and imaged under a fluorescence microscope using the 488 nm (green, monomeric form) and 555 nm (red, J-aggregate form) channels. Quantitative analysis was performed using ImageJ software. For each oocyte, a circle was drawn to outline the entire fluorescent region of the oocyte, and the same circle was used for measuring both red and green fluorescence intensities for that oocyte. The fluorescence intensities of red and green were measured separately, and MMP was expressed as the red-to-green fluorescence ratio. JC-1 forms red-fluorescent J-aggregates in mitochondria with high membrane potential. In contrast, it exists as green-fluorescent monomers when the membrane potential is low. MMP serves as the driving force for ATP synthesis via oxidative phosphorylation. A higher red/green ratio reflects greater mitochondrial functional integrity, while a lower ratio indicates impaired energy metabolism.

### 2.8. Measurement of Intracellular ATP Levels

Intracellular ATP levels in oocytes were measured using a BODIPY™ FL ATP assay kit (Invitrogen, USA) according to the manufacturer’s instructions. Oocytes were fixed and permeabilized with 4% paraformaldehyde containing 0.5% (*v*/*v*) Triton X-100 at 37 °C for 30 min. After three washes with 0.1% PVA/DPBS, oocytes were incubated with 5 μM BODIPY™ FL ATP at 38.5 °C in the dark for 30 min. Following three additional washes with 0.1% PVA/DPBS, the stained oocytes were immediately visualized and photographed under a laser confocal microscope (TCS SP8, Leica, Germany). Fluorescence intensity was quantified using ImageJ software (NIH, Bethesda, MD, USA).

### 2.9. scRNA-Seq Library Preparation and Sequencing Procedures

scRNA-seq libraries were prepared using a modified Smart-seq2 protocol [20,21]. Briefly, individual oocytes were picked with a mouth-drawn pipette and transferred into PCR tubes containing single-cell lysis buffer (0.1 U/μL RNase inhibitor, Triton X-100, dNTP mixture, and barcoded reverse transcription primers containing unique molecular identifiers (UMIs) lacking cytosine). After vigorous vortexing for 45 s, the samples were heated at 72 °C for 3 min to release linear RNA, immediately chilled on ice, and supplemented with reverse transcription mix (including SuperScript I reverse transcriptase, RNase inhibitor, first-strand synthesis buffer, betaine, MgCl_2_, TSO primer, and DTT). Reverse transcription was performed under the following conditions: 25 °C for 5 min, 42 °C for 60 min, 50 °C for 30 min, and 70 °C for 10 min, followed by holding at 4 °C.

Following reverse transcription, PCR reaction mix (containing 2× KAPA HiFi HotStart ReadyMix, ISPCR oligo, and 3′ Anchor oligo) was added for amplification. The thermal cycling program consisted of 4 cycles at 98 °C for 20 s, 65 °C for 15 s, and 72 °C for 5 min; 10–15 cycles at 98 °C for 20 s, 67 °C for 15 s, and 72 °C for 5 min; with a final extension at 72 °C for 5 min, followed by holding at 4 °C. Samples with different barcodes were pooled, purified using a DNAClean & Concentrator-5 kit, eluted in 50 μL H_2_O, and further purified with 0.8× Ampure XP beads. Purified cDNA was subjected to a second round of amplification (4–5 cycles) using biotinylated primers containing sequencing adapters and indices together with ISPCR oligo. The amplicons were purified with 0.8× Ampure XP beads and sheared to approximately 300 bp using a Covaris instrument (Brighton, UK). cDNA fragments carrying barcodes and UMIs were enriched with Dynabeads MyOne Streptavidin beads, and libraries were constructed using KAPA Hyper Prep Kits, ligated with NEB U-shaped adapters, and amplified for 8–10 cycles with Illumina QP2 primer and universal short primer. The final libraries were sequenced on an Illumina X Plus platform (San Diego, CA, USA) with 150 bp paired-end reads.

Raw sequencing reads from pooled STRT-seq libraries were demultiplexed based on cell barcode information using UMI-tools (version 1.1.6). The sequencing reads were subsequently separated into individual single-cell datasets according to their corresponding barcode sequences (Supplementary material S1) using custom scripts. The processed reads were quantified against the bovine reference transcriptome (Bos taurus ARS-UCD1.2 cDNA) using kallisto (v0.46.0). The resulting gene expression matrix for all genes is provided in Supplementary material 2.

The original data presented in the study are openly available in the Genome Sequence Archive in National Genomics Data Center (GSA: CRA047246) at https://ngdc.cncb.ac.cn/gsa/browse/CRA047246 (accessed on 16 September 2026).

### 2.10. Statistical Analysis

All experiments were performed with at least three independent replicates, and the results were expressed as means ± standard error of the mean (SEM). Graphs were generated using GraphPad Prism 10 (GraphPad Software, Boston, MA, USA). Statistical analyses were performed using one-way analysis of variance (ANOVA), followed by Duncan’s multiple range test for multiple comparisons, with SPSS software version 26.0 (IBM Corp., Armonk, NY, USA). A value of *p* < 0.05 was considered statistically significant.

## 3. Results

### 3.1. Effects of PA and SAL Supplementation During IVM on Oocyte Developmental Potential

To establish a PA-induced lipotoxicity model, we first screened the optimal PA concentration (Figure 1A–C). Supplementation of 0.2 mM PA in the maturation medium significantly reduced embryonic development compared with the control group, as reflected by the rates of 2-cell embryos (67.24% ± 1.43% vs. 83.04% ± 3.10%), 8-cell embryos (40.18% ± 2.90% vs. 48.89% ± 2.67%), and blastocysts (18.80% ± 2.67% vs. 34.15% ± 1.03%; all *p* < 0.05). An even more pronounced decrease was observed at 0.4 mM PA (2-cell: 62.80% ± 3.28%; 8-cell: 22.90% ± 1.79%), with blastocyst rate dropping to 15.21% ± 1.39%. Based on these results and the need for a detectable rescue effect in subsequent experiments, 0.2 mM PA was selected for establishing the lipotoxicity model. We next determined the optimal concentration of SAL. As shown in Figure 1D–F, SAL promoted embryonic development in a dose-dependent manner. Compared with the control group (2-cell: 80.30% ± 0.83%; 8-cell: 47.55% ± 1.90%; blastocyst: 32.78% ± 2.11%), treatment with 1, 5, and 10 μM SAL all markedly increased the rates of 2-cell embryos (83.34% ± 1.10%, 88.22% ± 1.89%, and 85.98% ± 1.09%, respectively), 8-cell embryos (52.39% ± 0.96%, 76.49% ± 1.54%, and 57.83% ± 3.05%, respectively), and blastocysts (41.24% ± 1.13%, 45.35% ± 1.74%, and 39.69% ± 2.67%, respectively; all *p* < 0.05), with 5 μM showing the most prominent efficacy. However, when the SAL concentration was increased to 20 μM (2-cell: 71.79% ± 1.09%; 8-cell: 43.55% ± 2.23%; blastocyst: 23.92% ± 2.55%) or 40 μM (2-cell: 65.92% ± 0.83%; 8-cell: 41.70% ± 1.64%; blastocyst: 19.15% ± 1.04%), this promoting effect gradually diminished and even turned to inhibition, suggesting that excessive SAL may adversely affect embryonic development.

### 3.2. SAL Supplementation During IVM Rescued the Impairment of Oocyte Maturation Quality Caused by PA Exposure

To evaluate oocyte maturation quality, we further assessed both nuclear and cytoplasmic maturation parameters. Cytoplasmic maturation was evaluated by examining the fluorescence intensities of F-actin (Figure 2A,B) and cortical granules (CGs) (Figure 2C,D), both of which are positively correlated with cytoplasmic maturation status. The results showed that PA treatment significantly reduced the fluorescence intensities of F-actin and CGs compared with the control group, whereas SAL treatment alone markedly increased both indicators. Notably, the PA + SAL group exhibited fluorescence intensities comparable to those of the control group, indicating that PA induced cytoplasmic maturation defects, which were effectively reversed by SAL supplementation. Nuclear maturation was assessed by the rate of first polar body extrusion (Figure 2E). PA treatment (60.33% ±2.68%, *p* < 0.05) significantly suppressed polar body extrusion, while SAL treatment (88.52% ± 3.75%, *p* < 0.05) alone markedly promoted this process, and no significant difference was observed between the PA + SAL group (80.72% ± 3.26%) and the control group (80.45% ± 3.65%). These findings demonstrate that PA exposure significantly impairs both nuclear and cytoplasmic maturation of oocytes, and that SAL effectively ameliorates PA-induced oocyte maturation defects.

### 3.3. SAL Alleviated PA Induced Impairment of Embryo Developmental Potential and Quality

To further evaluate the remedial capacity of SAL against the decline in oocyte developmental competence induced by PA (Figure 3A,B), the rates of 2-cell (80.94% ± 4.62%), 8-cell (49.25% ± 3.34%), and blastocyst formation (30.08% ± 1.93%) in the PA + SAL treatment group were statistically analyzed. The results showed that the developmental rates at all stages in this group were not significantly different from those in the control group (2-cell: 80.29% ± 2.33%, 8-cell: 50.09% ± 1.86%, blastocyst: 31.42% ± 1.50%), suggesting that SAL can effectively reverse the inhibitory effect of PA on oocyte embryonic developmental potential. In terms of blastocyst quality (Figure 3C), the total cell number (Figure 3D), inner cell mass (ICM) count (Figure 3E), and trophectoderm (TE) cell number (Figure 3F) were further assessed. The results demonstrated that PA treatment alone significantly reduced all the above parameters, whereas SAL treatment alone significantly enhanced the blastocyst quality-related indicators. Compared with the PA group, all parameters in the PA + SAL combined treatment group were significantly restored and showed no significant differences from the control group. These findings indicate that SAL not only alleviates the PA-induced blockade of embryonic developmental progression but also effectively improves blastocyst quality, exerting a clear protective and restorative effect.

### 3.4. Regulation of Gene Expression by SAL During Oocyte Maturation

To explore the role of SAL in oocyte maturation, we performed single-cell transcriptome analysis. We analyzed GV-stage oocytes, control MII oocytes and SAL-treated MII oocytes. Cell clustering showed that GV-stage oocytes had different transcriptional characteristics from the two MII groups (Figure 4A,B). Differential gene analysis revealed large transcriptomic changes during oocyte maturation. Compared with GV oocytes, the control group had 519 upregulated genes and 384 downregulated genes (Figure 4C). The SAL group had 356 upregulated genes and 500 downregulated genes (Figure 4D). In comparison with the control group, SAL treatment upregulated 72 genes and downregulated 387 genes (Figure 4E). To screen core SAL-responsive genes, we intersected upregulated genes among the three groups. Finally, 42 genes were identified that were highly expressed specifically in SAL-treated MII oocytes (Figure 4F). Functional enrichment showed that these genes were mainly enriched in RNA metabolism, protein homeostasis, cytoskeleton construction and basic metabolism (Figure 4G,H). These findings indicate that SAL improves oocyte physiological homeostasis by synergistically regulating multiple biological pathways.

### 3.5. SAL Effectively Rescued PA Induced Gene Expression Disorders

Next, to determine whether SAL could reverse PA-induced gene expression disorders in oocytes at the transcriptomic level, the transcriptomic data of oocytes were systematically compared. The results showed that the PA-treated group exhibited a distinct separate clustering, whereas the control group and the PA + SAL co-treatment group largely overlapped in sample clustering, suggesting that SAL effectively restored the transcriptomic shift induced by PA (Figure 5A). At the level of differentially expressed genes, compared with the control group, PA treatment resulted in 129 downregulated and 559 upregulated genes (Figure 5B); whereas the PA + SAL co-treatment group, relative to the PA group, showed 442 downregulated and 79 upregulated genes (Figure 5C). More importantly, when comparing the PA + SAL group with the control group, only 8 genes were downregulated and 23 genes were upregulated (Figure 5D), further indicating that the transcriptomic status returned to near-normal levels after SAL intervention. On this basis, the intersection of genes upregulated in the PA group compared with the control group and genes downregulated in the PA + SAL group compared with the PA group yielded 325 genes whose expression was restored to control levels (Figure 5E). Similarly, the intersection of genes downregulated in the PA group compared with the control group and genes upregulated in the PA + SAL group compared with the PA group yielded 55 genes that were also rescued (Figure 5F). These two intersection results demonstrate that SAL can effectively reverse the majority of gene expression disturbances caused by PA.

### 3.6. SAL Attenuated PA Induced Mitochondrial Dysfunction

To explore the potential functions of the genes downregulated by PA treatment, we performed pathway enrichment analysis and found that these genes were significantly enriched in mitochondrial function related pathways (Figure 6A). As shown in Figure 6B, multiple genes involved in oxidative phosphorylation and ATP synthesis (including *NDUFA4*, *NDUFA6*, *ATP5PO*, *ATP5ME*, *COX5A*, and *UQCRH*) were markedly downregulated after PA treatment, whereas SAL intervention substantially upregulated their expression. To validate these transcriptomic findings, we measured mitochondrial membrane potential (Figure 6C,D) and endogenous ATP content (Figure 6E,F), and observed that PA treatment led to a significant reduction in both parameters, while SAL treatment effectively restored their levels. Thus, these results indicate that PA induces energy metabolism disorders in oocytes by suppressing the expression of mitochondrial function related genes, and SAL can effectively rescue this impairment.

### 3.7. SAL Protected Oocytes Against PA-Induced Oxidative Damage

Oxidative stress is a major contributor to mitochondrial dysfunction. To this end, we further analyzed the expression changes in oxidative stress-related genes in the transcriptomic data. The results showed that after PA treatment, the expression levels of *HSPE1*, *ROMO1*, and *GSTM3* were significantly reduced, whereas SAL intervention effectively restored their expression to approximately normal levels (Figure 7A). Meanwhile, measurement of ROS (Figure 7B,C) and GSH (Figure 7B,D) contents revealed that PA treatment markedly increased intracellular ROS levels and decreased GSH content in oocytes, while SAL treatment reversed these changes. These results indicate that PA induces oxidative damage in oocytes, further supporting its disruptive effect on mitochondrial function, and that SAL can effectively alleviate such damage.

## 4. Discussion

Here, we employed PA to mimic the high-lipid microenvironment of follicular fluid and found that PA accumulation provoked oxidative stress in oocytes, culminating in a significant impairment of their developmental competence. Notably, supplementation with SAL effectively attenuated PA-induced oxidative stress, restored mitochondrial function, and ultimately improved the developmental capacity of oocytes and their derived embryos.

PA, one of the most abundant saturated fatty acid in follicular fluid, has been recognized as a critical pathogenic factor mediating lipotoxic insults to the female reproductive system [10,22]. Elevated PA levels are not only observed in dairy cows under negative energy balance [23], but also frequently detected in the follicular fluid of infertile women with metabolic disorders, including obesity [24], polycystic ovary syndrome [25], and insulin resistance [26]. The excessive accumulation of PA disrupts the homeostatic equilibrium of the follicular fluid microenvironment, thereby imposing a profound detrimental impact on oocyte developmental competence [27]. In alignment with this, our in vitro study demonstrated that supplementation of 0.2 mM PA during oocyte maturation markedly impaired oocyte maturation, subsequent embryo development, and blastocyst quality. PA triggered oxidative stress in oocytes and subsequently caused mitochondrial dysfunction, which is in agreement with earlier observations [28,29,30,31].

Mitochondria are the most abundant organelles in oocytes, and their functional status is closely linked to oocyte maturation, fertilization, and early embryonic developmental potential [32,33,34]. MMP serves as a critical indicator of mitochondrial function, the maintenance of which relies on the coordinated activity of respiratory chain complexes I–V [35,36]. In the present study, treatment with palmitic acid (PA) significantly downregulated multiple respiratory chain related genes in oocytes, including *NDUFA4*, *NDUFA6*, *UQCRH*, *COX5A*, *ATP5PO*, and *ATP5ME*, which are involved in electron transfer initiation, Q cycle mediation, oxygen reduction, and ATP synthesis, respectively. The broad suppression of these genes at the transcriptional level provides a mechanistic explanation for the PA induced decline in MMP and ATP content [7,37]. Previous studies have also demonstrated that impairment of the mitochondrial oxidative phosphorylation pathway severely compromises oocyte quality and reduces blastocyst formation rate [38,39], which is consistent with our findings that PA disrupts electron transport chain function, diminishes ATP production, and ultimately impairs oocyte quality.

SAL is a natural multi-target compound with well-documented antioxidant and cytoprotective activities [40,41,42]. Previous studies have reported its beneficial effects on oocyte quality [17], embryonic development, and cardiovascular protection [43]. In the present study, supplementation with 5 μM SAL during IVM significantly promoted both nuclear and cytoplasmic maturation of bovine oocytes under non-stressed conditions, and notably enhanced subsequent embryonic developmental potential and blastocyst quality. Single-cell transcriptomic analysis further revealed that SAL considerably upregulated genes such as *DNAJC2*, *OPTN*, and *PSMA7*, which are integral to RNA processing, translational regulation, the ubiquitin-proteasome system, mitophagy, and cytoskeletal assembly. These observations suggest that SAL may influence multiple functional modules, rather than acting through a single pathway. However, given that IVM, IVF, and IVP were all performed under 20% O_2_—a pro-oxidative atmosphere that promotes ROS generation—the control oocytes and embryos were already cultured under a basal state of oxidative stress. The protective effects of SAL can be primarily attributed to the preservation of cellular antioxidant potential under these conditions. The transcriptomic changes associated with RNA processing, protein homeostasis, mitophagy, and cytoskeletal regulation may represent secondary or adaptive responses, and their causal roles in mediating SAL’s protective effects remain to be experimentally validated [44,45,46,47,48,49,50,51]. Based on these findings, we propose that SAL enhances oocyte developmental competence primarily by preserving cellular antioxidant capacity, and it may additionally coordinate a broader transcriptomic response that synergistically optimizes multiple cellular functions.

Furthermore, PA treatment provoked oxidative stress in oocytes, as evidenced by elevated ROS levels and reduced GSH content, along with transcriptional suppression of *HSPE1*, *ROMO1*, and *GSTM3*. *HSPE1* serves as a key guardian of mitochondrial protein homeostasis, and its downregulation increases the burden of protein misfolding, thereby threatening mitochondrial functional integrity [52,53]. *ROMO1* downregulation leads to mitochondrial fragmentation and dissipation of membrane potential, which further aggravates energy metabolic failure [54]. Meanwhile, reduced *GSTM3* expression impairs glutathione dependent detoxification, lowering the cellular threshold for defending against oxidative attacks [55,56,57,58]. These data indicate that PA does not merely compromise mitochondrial energy metabolism. Instead, PA dismantles the coordinated stress defense network, driving mitochondria toward collapse under the dual pressure of energy crisis and oxidative injury. SAL intervention effectively reverses this gene expression pattern. It restores respiratory chain function and reactivates antioxidant defense systems, thereby enhancing oocyte tolerance and repair capacity under stressful conditions. This multi-target restorative effect, rather than single pathway compensation, aligns well with the natural properties of SAL as a multifunctional molecule and provides a mechanistic explanation for its ability to rescue oocyte developmental competence at the transcriptional level.

## 5. Conclusions

In conclusion, PA impairs oocyte developmental potential, most likely by disrupting redox balance, although this requires further validation. Considering the 20% O_2_ environment during IVP, the protective effect of SAL in restoring oxidative homeostasis and improving oocyte quality is particularly notable, as PA acts as an additional stressor on top of the basal oxygen-induced challenge. These findings support SAL as a practical intervention for improving oocyte quality in dairy cows, but also offer a novel theoretical basis for improving oocyte developmental competence under lipotoxic microenvironmental conditions.

## Figures and Tables

**Figure 1:**
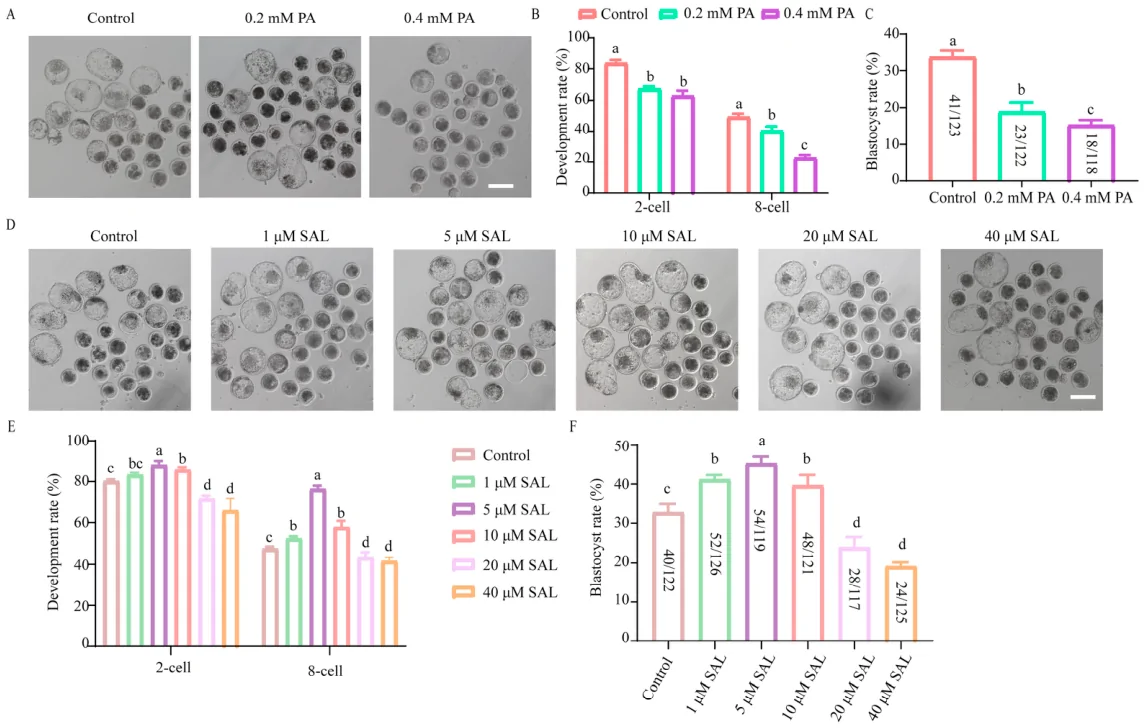
Effects of different concentrations of PA and SAL on embryonic development. (**A**) Representative images blastocysts of the PA-treated and control groups, scale bar = 200 μm. (**B**) Developmental rates of the PA-treated and control groups (2-cell rates: replicates = 4, cleaved embryos/zygote; control, 102/123; 0.2 mM PA, 82/122; 0.4 mM PA, 74/118) (8-cell rates: replicates = 4, 8-cell embryos /zygote; control, 60/123; 0.2 mM PA, 49/122; 0.4 mM PA, 27/118). (**C**) Blastocyst rates of the PA-treated and control groups (Blastocyst rates: replicates = 4, blastocyst s /zygote; control, 42/123; 0.2 mM PA, 23/122; 0.4 mM PA, 18/118). (**D**) Representative images of blastocysts in the SAL-treated group, scale bar = 200 μm. (**E**) Developmental rates of the SAL-treated and control groups (2-cell rates: replicates = 4, cleaved embryos/zygote; control, 98/122; 1 μM SAL, 105/126; 5 μM SAL, 105/119; 10 μM SAL, 104/121; 20 μM SAL, 84/117; 40 μM SAL, 82/125) (8-cell rates: replicates = 4, 8-cell embryos /zygote; control, 58/122; 1 μM SAL, 66/126; 5 μM SAL, 91/119; 10 μM SAL, 70/121; 20 μM SAL, 51/117; 40 μM SAL, 52/125). (**F**) Blastocyst rates of the SAL-treated and control groups (Blastocyst rates: replicates = 4, blastocyst s /zygote; control, 40/122; 1 μM SAL, 52/126; 5 μM SAL, 54/119; 10 μM SAL, 48/121; 20 μM SAL, 28/117; 40 μM SAL, 24/125). The experiments were repeated at least three times. Different superscript letters (a–d) indicate statistically significant differences (*p* < 0.05).

**Figure 2:**
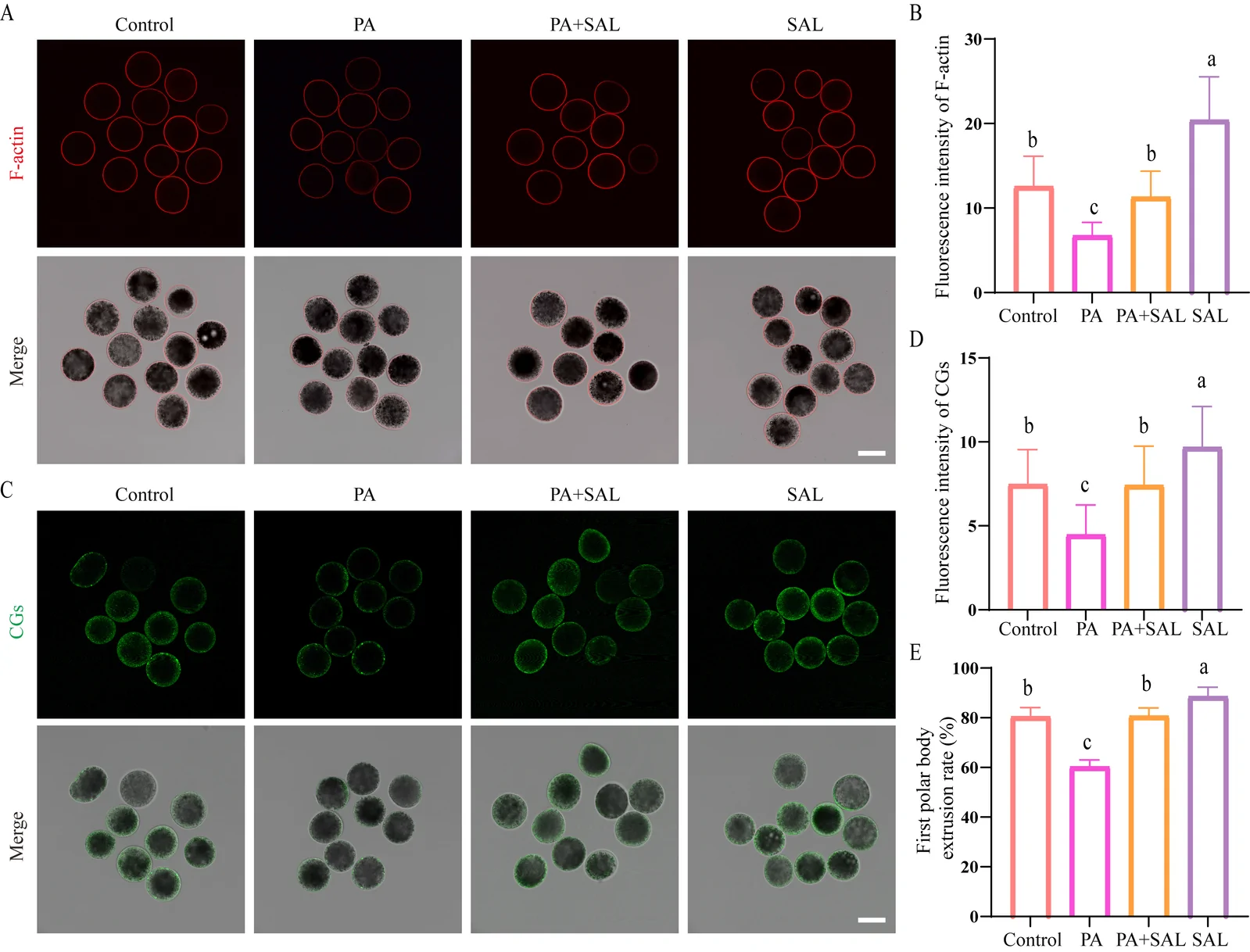
Assessment of oocyte maturation status. (**A**) Representative images of F-actin staining (Red fluorescence indicates F-actin filaments), scale bar = 100 μm. (**B**) Statistics of F-actin fluorescence intensity (replicates = 3, denuded oocytes : control, *n* = 29; PA, *n* = 28; PA + SAL, *n* = 31; SAL, *n* = 33). (**C**) Representative images of cortical granule (CGs) staining (Green fluorescence indicates cortical granules), scale bar = 100 μm. (**D**) Statistics of CGs fluorescence intensity (replicates = 3, denuded oocytes: control, *n* = 32; PA, *n* = 30; PA + SAL, *n* = 33; SAL, *n* = 30). (**E**) Statistics of first polar body extrusion rate in oocytes (replicates = 4, MII oocytes/COCs; control, 107/133; PA, 86/142; PA + SAL, 115/142; SAL, 132/148). Different superscript letters (a–c) indicate statistically significant differences (*p* < 0.05).

**Figure 3:**
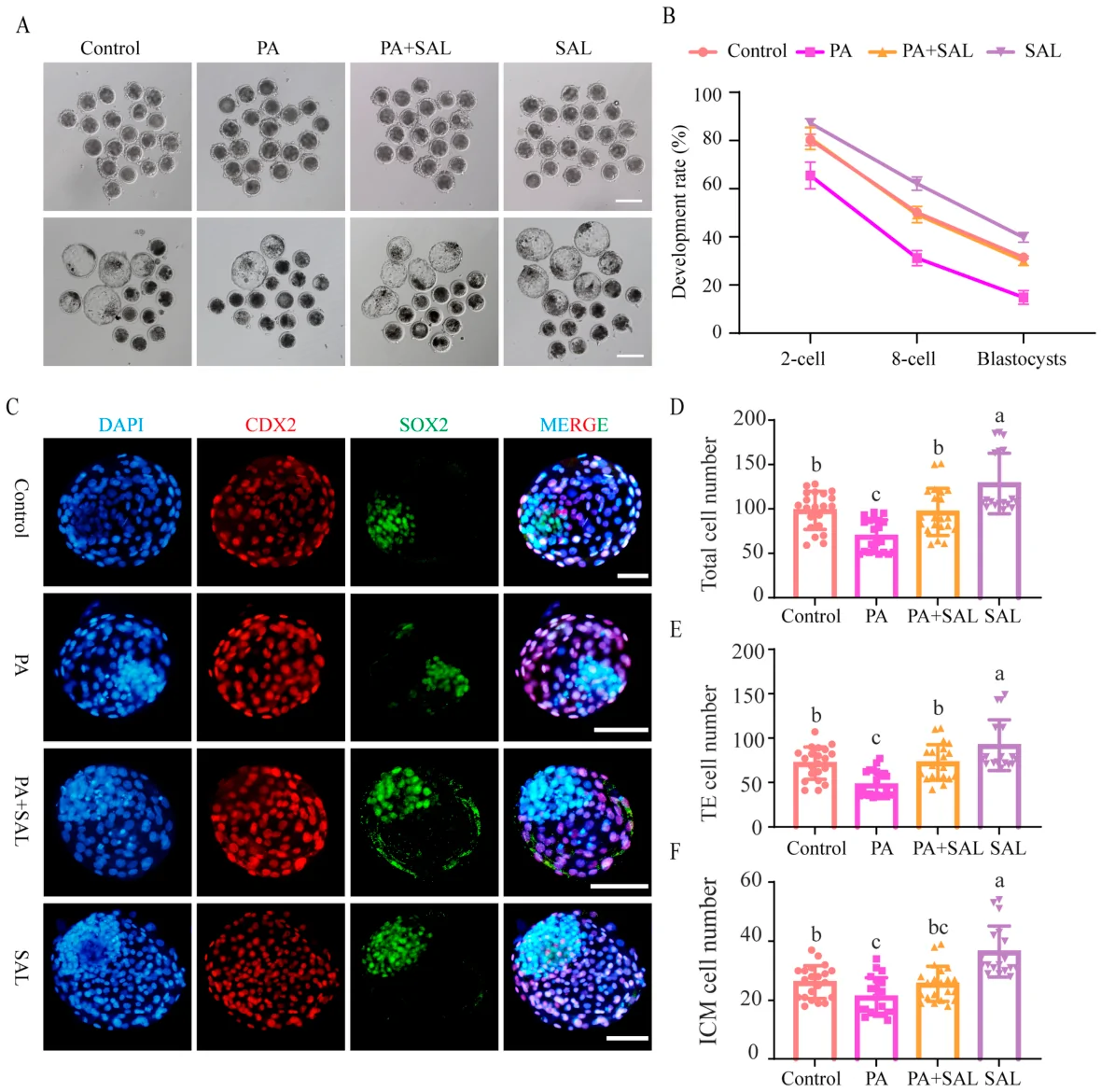
Evaluation of blastocyst developmental efficiency and quality. (**A**) Representative images of cleaved embryos and blastocysts in different treatment groups, scale bar = 200 μm. (**B**) Statistics of embryonic developmental efficiency (2-cell rates: replicates = 3, cleaved embryos/zygote; control, 94/118; PA, 84/125; PA + SAL, 101/127; SAL, 103/119) (8-cell rates: replicates = 3, 8-cell embryos /zygote; control, 59/118; PA, 38/125; PA + SAL, 64/127; SAL, 75/119) (Blastocyst rates: replicates = 3, blastocyst s /zygote; control, 37/118; PA, 18/125; PA + SAL, 38/127; SAL, 48/119). (**C**) Representative images of blastocyst labeling with DAPI (blue), SOX2 antibody (green), and CDX2 antibody (red), scale bar = 100 μm. (**D**) Total cell number. (**E**) Trophectoderm (TE) cell number. (**F**) Inner cell mass (ICM) cell number (replicates = 3, blastocysts, *n*; control, *n* = 24; PA, *n* = 20; PA + SAL, *n* = 21; SAL, *n* = 23). Different superscript letters (a–c) indicate statistically significant differences (*p* < 0.05).

**Figure 4:**
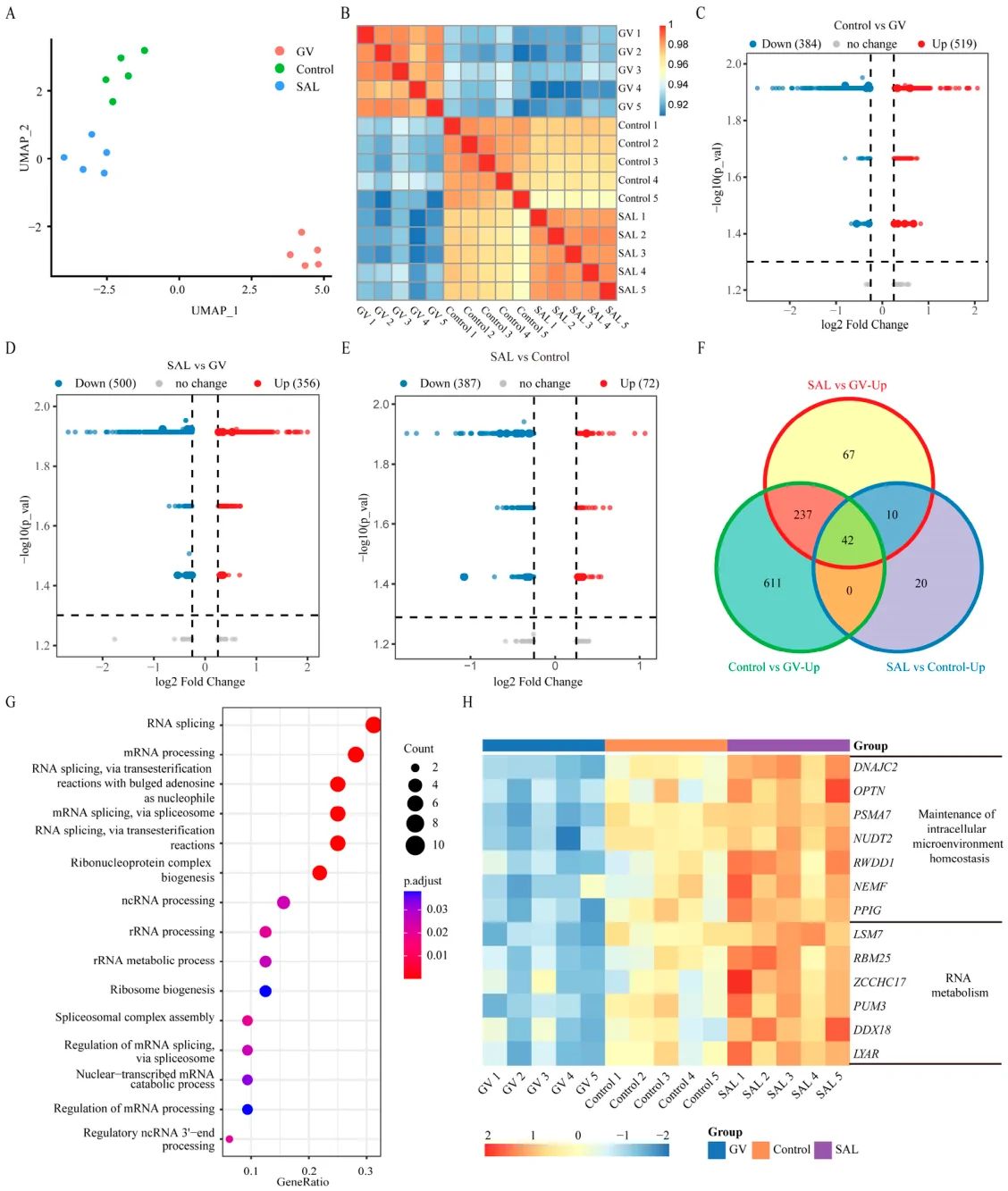
Single-cell transcriptomic analysis reveals the regulatory role of SAL in oocyte maturation. (**A**) UMAP plot of single-cell transcriptomes from GV stage oocytes, control (MII oocytes), and SAL (MII oocytes). Each dot represents a single cell, and colors indicate different groups. (**B**) Correlation analysis among different experimental groups. (**C**) Volcano plot of differentially expressed genes between the control and GV groups. Red and blue dots represent upregulated and downregulated genes, respectively (|log_2_FC| ≥ 0.25, *p* < 0.05). Gray dots indicate genes with no significant change. (**D**) Volcano plot of differentially expressed genes between the SAL and GV groups. (**E**) Volcano plot of differentially expressed genes between the control and SAL groups. (**F**) Venn diagram showing the overlap of differentially expressed genes among SAL vs. GV (upregulated genes), Control vs. GV (upregulated genes), and SAL vs. control (upregulated genes). The numbers represent the count of genes shared between or unique to each comparison. (**G**) GO enrichment analysis based on the 42 overlapping genes identified in F. Bubble size represents the number of genes enriched in each GO term, and the color gradient reflects the significance level of enrichment. (**H**) Heatmap showing the expression of genes involved in RNA metabolism and intracellular homeostasis maintenance across different treatment groups. Each column represents a sample, each row represents a gene, and the color scale from blue to red indicates expression levels from low to high.

**Figure 5:**
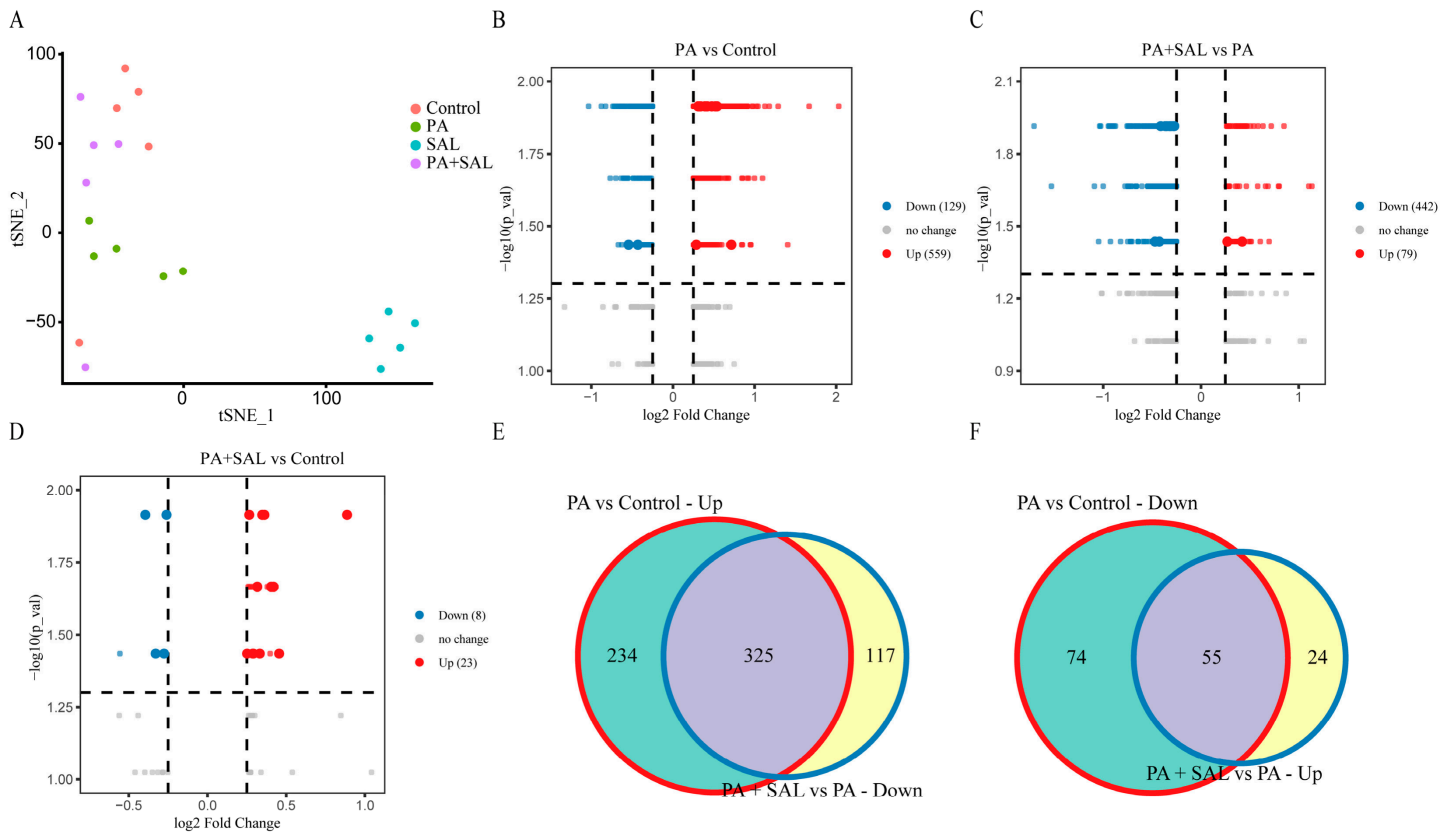
SAL reverses PA-induced transcriptomic alterations in bovine oocytes. (**A**) t-SNE analysis of transcriptomic profiles of oocytes across different treatment groups. Each dot represents a single-cell sample, and different colors indicate distinct experimental groups (Control, PA, SAL, and PA + SAL groups). The distance between points in the two-dimensional t-SNE space reflects the transcriptional similarity among samples, with closer distances indicating more similar expression profiles. (**B**) Volcano plot of differentially expressed genes between the PA- and control-treated groups. Red and blue dots represent significantly upregulated and downregulated genes, respectively (|log_2_FC| ≥ 0.25, *p* < 0.05). (**C**). Volcano plot of differentially expressed genes between the PA + SAL-treated and PA groups. (**D**). Volcano plot of differentially expressed genes between the PA + SAL and control groups. (**E**). Venn diagram showing the intersection (*n* = 325) of genes upregulated in the PA group compared with the control group and genes downregulated in the PA + SAL group compared with the PA group, representing PA upregulated genes reversed by SAL. (**F**). Venn diagram showing the intersection (*n* = 55) of genes downregulated in the PA group compared with the control group and genes upregulated in the PA + SAL group compared with the PA group, representing PA downregulated genes restored by SAL.

**Figure 6:**
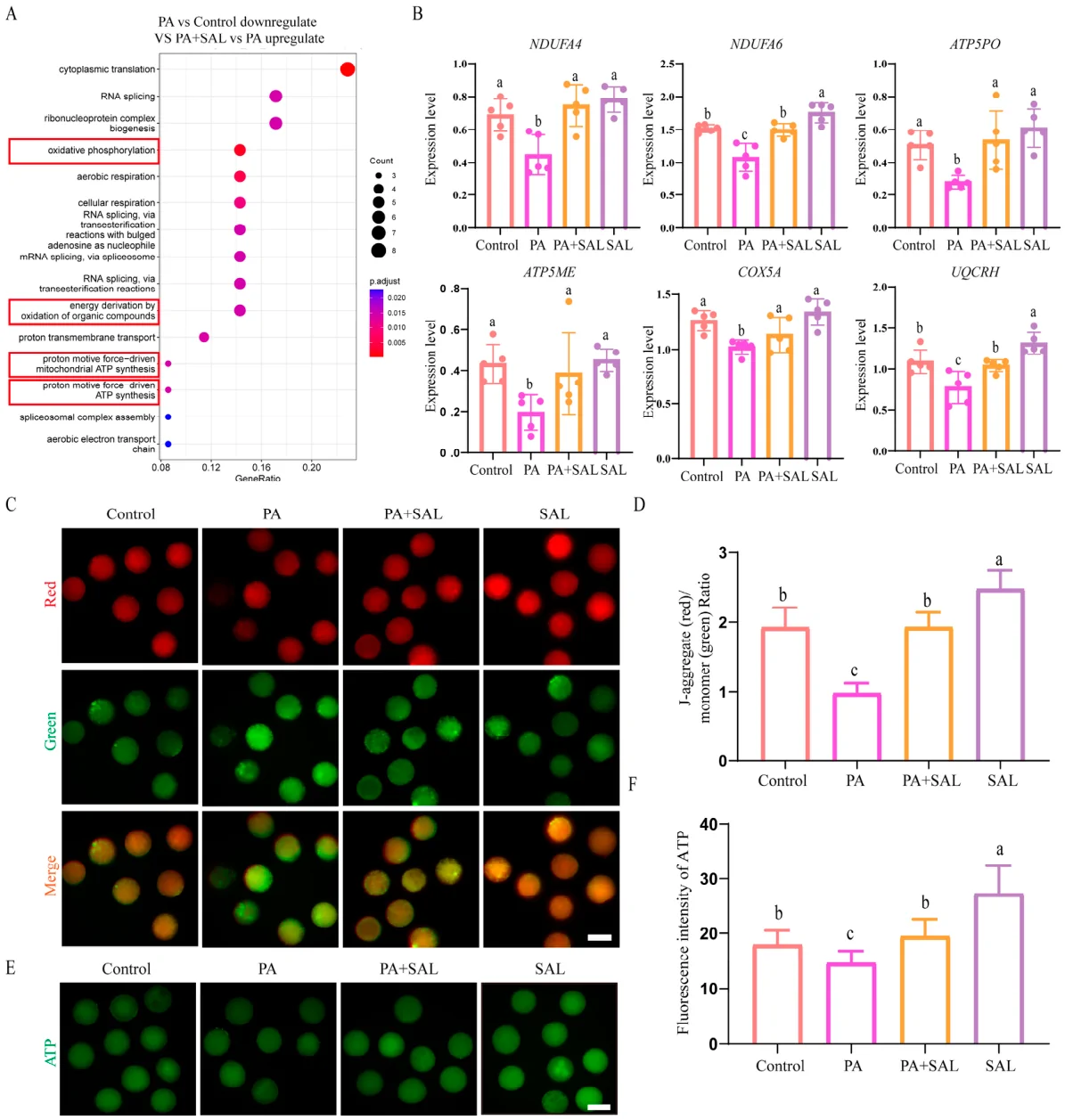
Rescue effect of SAL on PA induced mitochondrial damage in oocytes. (**A**) GO enrichment analysis based on the 55 overlapping genes identified in Figure 5F (Red box: mitochondria-related signalling pathway). (**B**) Expression levels of mitochondrial function related genes across different treatment groups. (**C**) Representative images of MMP in bovine oocytes stained with JC-1 dye, scale bar = 100 μm. (**D**) Relative MMP was expressed as the ratio of red (JC 1 aggregates, high membrane potential) to green (JC 1 monomers, low membrane potential) fluorescence intensity (replicates = 3, denuded oocytes, *n*; control, *n* = 28; PA, *n* = 32; PA + SAL, *n* = 31; SAL, *n* = 29). (**E**) Representative images of ATP in oocytes stained with BODIPY™ FL ATP dye, scale bar = 100 μm. (**F**) Statistics of relative ATP fluorescence intensity (replicates = 3, denuded oocytes, *n*; control, *n* = 36; PA, *n* = 27; PA + SAL, *n* = 33; SAL, *n* = 31). Different superscript letters (a–c) indicate statistically significant differences (*p* < 0.05).

**Figure 7:**
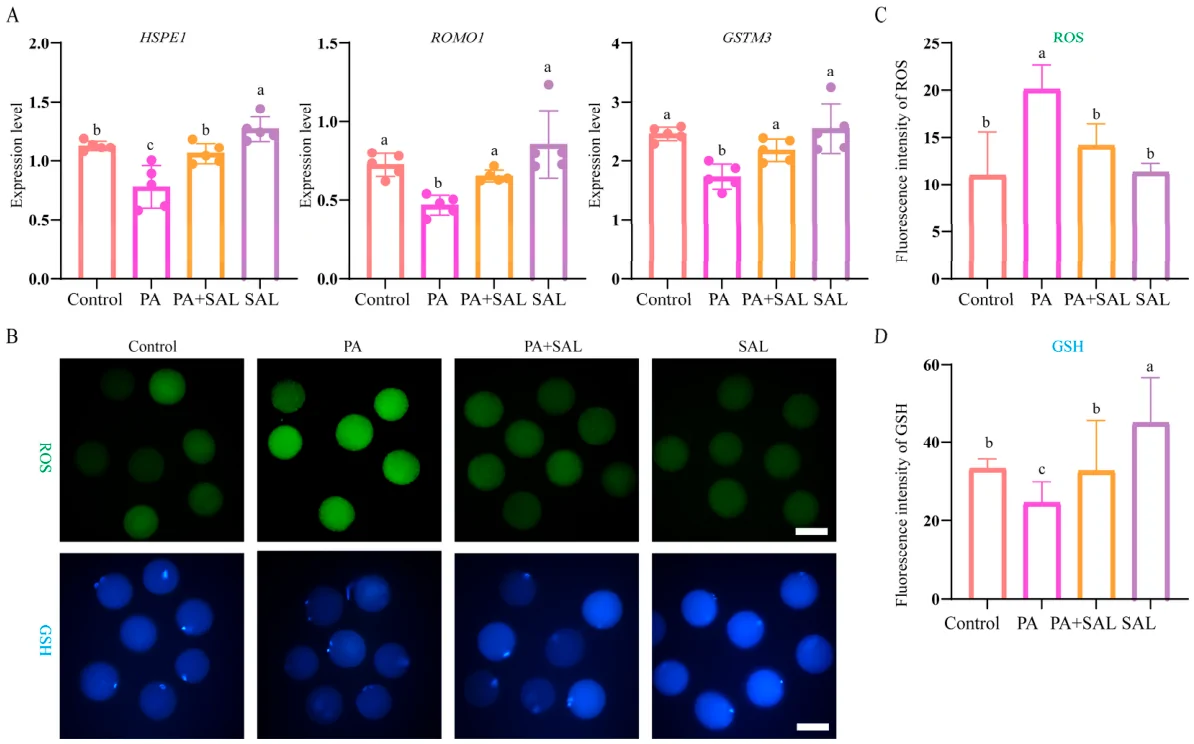
Rescue effect of SAL on PA-induced oxidative stress in oocytes. (**A**) Expression levels of oxidative stress-related genes across different treatment groups. (**B**) Representative images of intracellular ROS and GSH levels in oocytes stained with DCHFDA and ThiolTracker™ Violet, respectively, scale bar = 100 μm. (**C**) Relative ROS fluorescence intensity (replicates = 3, denuded oocytes, *n*; control, *n* = 28; PA, *n* = 27; PA + SAL, *n* = 24; SAL, *n* = 27). (**D**) Relative GSH fluorescence intensity (replicates = 3, denuded oocytes, *n*; control, *n* = 30; PA, *n* = 28; PA + SAL, *n* = 25; SAL, *n* = 25). Different superscript letters (a–c) indicate statistically significant differences (*p* < 0.05).

## Data Availability

The original data presented in the study are openly available in the Genome Sequence Archive in National Genomics Data Center (accession number GSA: CRA047246), which are publicly accessible at https://ngdc.cncb.ac.cn/gsa/browse/CRA047246 (accessed on 16 September 2026).

## Supplementary Materials

The following supporting information can be downloaded at: https://www.mdpi.com/article/10.3390/antiox15101254/s1. Supplementary material S1: supplementary data_scRNAseq_barcode; Supplementary material S2: Gene_expression_all_groups.
